# *fac*-[1,2-Bis(pyridin-4-yl)ethane-κ*N*]tricarbon­yl(1,10-phenanthroline-κ^2^ *N*,*N*′)rhenium(I) hexa­fluorido­phosphate aceto­nitrile monosolvate

**DOI:** 10.1107/S1600536814014135

**Published:** 2014-06-25

**Authors:** Silvana Guilardi, Antonio Otavio Toledo Patrocinio, Sinval Fernandes de Sousa, Javier Ellena

**Affiliations:** a Instituto de Química – UFU, Uberlândia–MG, Brazil; b Instituto de Física de São Carlos – USP, 13500-970–São Carlos, SP, Brazil

**Keywords:** crystal structure

## Abstract

The asymmetric unit of the title compound, [Re(C_12_H_8_N_2_)(C_12_H_12_N_2_)(CO)_3_]PF_6_.·CH_3_CN, contains one cation, one hexa­fluorido­phosphate anion and one aceto­nitrile solvent mol­ecule. The Re^I^ ion is coordinated by two N atoms from the 1,10-phenanthroline ligand and one N atom from the 1,2-bis­(pyridin-4-yl)ethane ligand [mean Re—N = 2.191 (15) Å] and by three carbonyl ligands [mean Re—C = 1.926 (3) Å] in a distorted octa­hedral geometry. The electrostatic forces and weak C—H⋯F(O) hydrogen bonds pack cations and anions into the crystal with voids of 82 Å^3^, which are filled by solvent mol­ecules. The crystal packing exhibits short inter­molecular O⋯O distance of 2.795 (5) Å between two cations related by inversion.

## Related literature   

For photophysical and photochemical properties of rhenium(I)–polypyridyl complexes, see: Li *et al.* (2012 ▶); Mizoguchi *et al.* (2009 ▶); Patrocinio *et al.* (2010 ▶, 2013 ▶); Thorp-Greenwood *et al.* (2012 ▶). For similar compounds and their crystal structures, see: Ranjan *et al.* (2003 ▶); Wenger *et al.* (2004 ▶); Ide *et al.* (1995 ▶). For details of the synthetic procedure, see: Patrocinio *et al.* (2010 ▶); Patrocinio & Murakami Iha (2008 ▶); Argazzi *et al.* (2001 ▶).
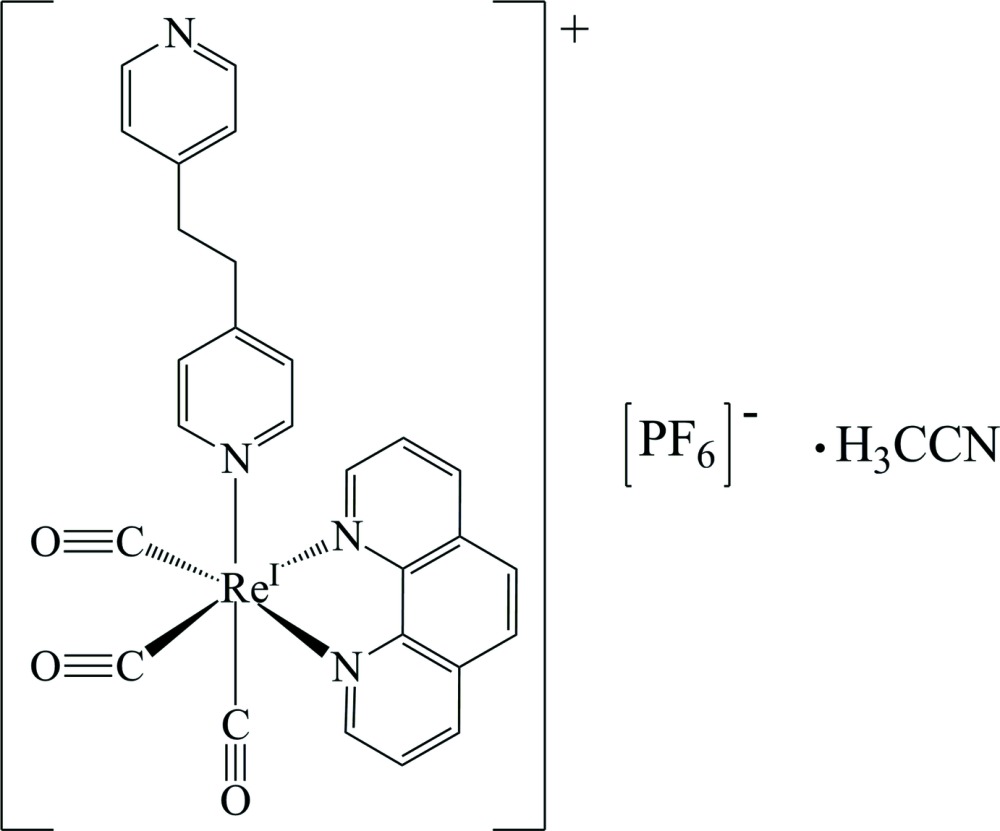


## Experimental   

### 

#### Crystal data  

[Re(C_12_H_8_N_2_)(C_12_H_12_N_2_)(CO)_3_]PF_6_·C_2_H_3_N
*M* *_r_* = 820.69
Monoclinic, 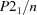

*a* = 10.5992 (2) Å
*b* = 16.1201 (3) Å
*c* = 17.3449 (2) Å
β = 100.879 (1)°
*V* = 2910.29 (8) Å^3^
*Z* = 4
Mo *K*α radiation
μ = 4.31 mm^−1^
*T* = 100 K
0.29 × 0.20 × 0.13 mm

#### Data collection  

Nonius KappaCCD diffractometer
Absorption correction: Gaussian (Coppen *et al.*, 1965 ▶) *T*_min_ = 0.386, *T*_max_ = 0.613
35034 measured reflections
6066 independent reflections
5415 reflections with *I* > 2σ(*I*)
*R*_int_ = 0.106

#### Refinement  

*R*[*F* ^2^ > 2σ(*F* ^2^)] = 0.041
*wR*(*F* ^2^) = 0.109
*S* = 1.09
6066 reflections
406 parameters
H-atom parameters constrained
Δρ_max_ = 1.39 e Å^−3^
Δρ_min_ = −2.50 e Å^−3^

### 

Data collection: *COLLECT* (Hooft, 2004 ▶); cell refinement: *SCALEPACK* (Otwinowski & Minor, 1997 ▶); data reduction: *DENZO* (Otwinowski & Minor 1997 ▶) and *SCALEPACK*; program(s) used to solve structure: *SHELXS97* (Sheldrick, 2008 ▶); program(s) used to refine structure: *SHELXL97* (Sheldrick, 2008 ▶); molecular graphics: *ORTEP-3 for Windows* (Farrugia, 2012 ▶); software used to prepare material for publication: *WinGX* (Farrugia, 2012 ▶).

## Supplementary Material

Crystal structure: contains datablock(s) I, global. DOI: 10.1107/S1600536814014135/cv5465sup1.cif

Structure factors: contains datablock(s) I. DOI: 10.1107/S1600536814014135/cv5465Isup2.hkl

CCDC reference: 1008626

Additional supporting information:  crystallographic information; 3D view; checkCIF report

## Figures and Tables

**Table 1:** Hydrogen-bond geometry (Å, °)

*D*—H⋯*A*	*D*—H	H⋯*A*	*D*⋯*A*	*D*—H⋯*A*
C13—H13⋯F4	0.93	2.47	3.377 (6)	166
C16—H16⋯F6	0.93	2.38	3.180 (6)	145
C11—H11⋯F5^i^	0.93	2.55	3.327 (6)	142
C12—H12⋯F1^i^	0.93	2.55	3.355 (6)	145
C5—H5⋯F1^ii^	0.93	2.52	3.382 (6)	154
C19—H19⋯F4^iii^	0.93	2.49	3.150 (6)	128
C20—H20⋯F5^iii^	0.93	2.45	3.317 (6)	154
C21—H21*A*⋯F5^iv^	0.97	2.53	3.486 (6)	168
C22—H22*B*⋯O1^v^	0.97	2.53	3.211 (8)	127

Symmetry codes: (i) 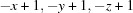; (ii) 
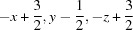
; (iii) 
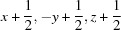
; (iv) 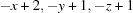; (v) 
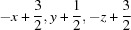
.

**Fig. 1.**
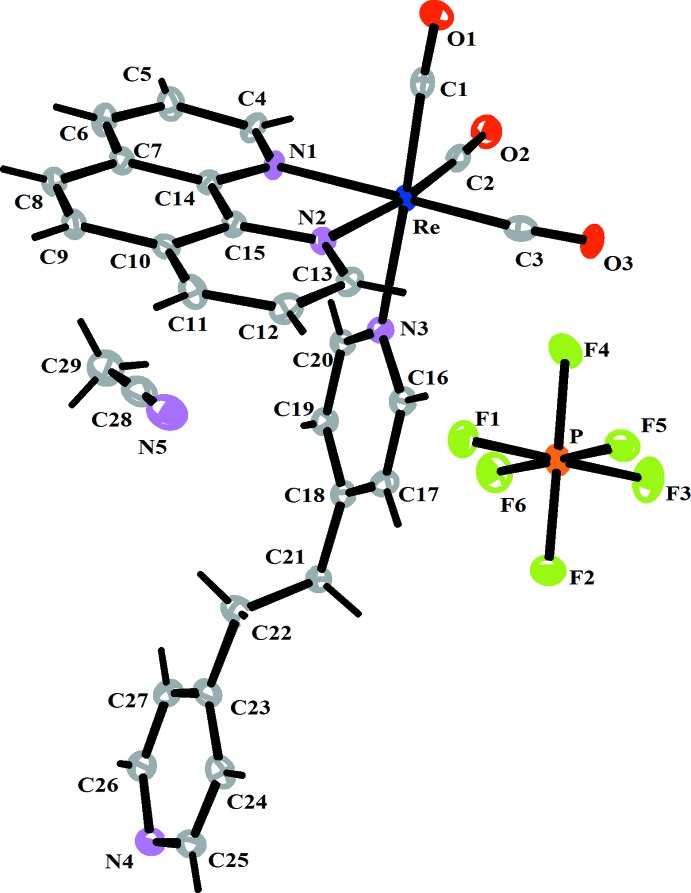
A view of the asymmetric unit of the title compound, showing the atom labeling and 30% probability displacement ellipsoids.

## supplementary crystallographic information

### S1. Comment

Rhenium(I) polypyridyl complexes exhibit very interesting photophysical and photochemical properties which can be exploited in the development of different photochemical molecular devices. Interesting examples can be found in the literature such as light emitting devices (Li *et al.*, 2012; Mizoguchi *et al.*, 2009), photoswitches (Patrocinio *et al.*, 2010, 2013), DNA sensors (Thorp-Greenwood *et al.*, 2012), among others. In this article, we describe the crystal structure of a Re^I^ polypyridyl complex having 1,2-bis(pyridin-4-yl)ethane (bpa) as ancillary ligand. This complex can be conveniently used as luminescent material and also as a construction unit for binuclear complex in intramolecular energy transfer studies.

The asymmetric unit in the title compound consists of the complex cation [Re(CO)_3_(phen)(bpa)]^+^ (phen = 1,10-phenanthroline), PF_6_^-^ anion and one acetonitrile solvent molecule (Fig. 1). The Re^I^ center has a distorted octahedral environment. It is coordinated by three carbonyl groups arranged in a facial fashion [mean Re–C distance of 1.926 (6) Å], two nitrogen atoms from phen ligand [mean Re–N distance of 2.183 (4) Å] and one nitrogen atom from bpa ligand [Re–N distance of 2.208 (4) Å]. The Re–C and Re–N distances were comparable to those of related systems (Ranjan *et al.*, 2003; Wenger *et al.*, 2004). The bidentate bite angle N–Re–N is 76.0 (2)°. The Re^I^ lies -0.055 (4) Å from the least-squares plane of 1,10-phenanthroline. In the bpa ligand, the bond distance C21–C22 is 1.526 (8) Å. The C21 ethane carbon atom is nearly coplanar with N3-pyridyl moiety. The C21–C22–C23–C24 and C21–C22–C23–C27 torsion angles are 102.5 (7)° and -75.2 (8)°, respectively. These angles in the free bpa ligand are 78.0 (3)° and -99.5 (3)°, respectively (Ide *et al.*, 1995). The bond lengths in the structure of the free bpa ligand are shorter than those observed in the ligand coordinated to the metallic center. The PF_6_^-^ anion adopts an octahedral geometry with P–F distances varied from 1.587 (4) to 1.612 (3) Å. The components of the structure are connected into a three-dimensional architecture by electrostatic forces and C—H···F and C—H···O hydrogen bonds (Table 1).

### S2. Experimental

The *fac*-[Re(CO)_3_(phen)(bpa)]PF_6_ compound (phen = 1,10-phenanthroline, bpa = 1,2-bis(4-pyridyl)ethane) was prepared following the procedures described earlier (Patrocinio *et al.*, 2010; Patrocinio & Murakami Iha, 2008; Argazzi *et al.*, 2001). Briefly, [ClRe(CO)_5_] and an excess of the polipyridyl ligand were refluxed in toluene for 5–7 h to yield a yellow solid, *fac*-[ClRe(CO)_3_(NN)]. The product was collected by filtration and recrystallized from CH_2_Cl_2_ by slow addition of n-pentane. Then, the *fac*-[ClRe(CO)_3_(NN)] complexes were suspended in argon-saturated CH_2_Cl_2_ and trifluoromethanesulfonic acid was added to reaction mixture to yield the respective intermediates *fac*-[Re(CO)_3_(NN)(CF_3_SO_3_)], which were precipitated by slow addition of ethyl ether. Finally, an excess of the bpa ligand were added to *fac*-[Re(CO)_3_(NN)(CF_3_SO_3_)] in methanol and the mixture were kept in reflux under argon atmosphere during 8–9 h. After cooling, the final products were obtained by addition of solid NH_4_PF_6_. The solids were separated by filtration, washed with water to remove the NH_4_PF_6_ excess and ethyl ether to dry *fac*-[Re(CO)_3_(phen)(bpa)]PF_6_ were crystallized by slow diffusion of diethyl ether into an acetonitrile solution at room temperature.

### S3. Refinement

H atoms were included in calculated positions (C–H = 0.93 Å for aromatic H, C–H = 0.97 Å for methylene H and C–H = 0.96 Å for methyl H), and refined using a riding model with *U*_iso_(H) = 1.2 or 1.5 *U*_eq_ of the carrier atom.

### Crystal data

**Table:**

[Re(C_12_H_8_N_2_)(C_12_H_12_N_2_)(CO)_3_]PF_6_·C_2_H_3_N	*F*(000) = 1600
*M**_r_* = 820.69	*D*_x_ = 1.873 Mg m^−^^3^
Monoclinic, *P*2_1_/*n*	Mo *K*α radiation, λ = 0.71073 Å
*a* = 10.5992 (2) Å	Cell parameters from 20552 reflections
*b* = 16.1201 (3) Å	θ = 2.9–26.7°
*c* = 17.3449 (2) Å	µ = 4.31 mm^−^^1^
β = 100.879 (1)°	*T* = 100 K
*V* = 2910.29 (8) Å^3^	Prism, colourless
*Z* = 4	0.29 × 0.20 × 0.13 mm

### Data collection

**Table:**

Nonius KappaCCD diffractometer	6066 independent reflections
Radiation source: Enraf–Nonius FR590	5415 reflections with *I* > 2σ(*I*)
Graphite monochromator	*R*_int_ = 0.106
Detector resolution: 9 pixels mm^-1^	θ_max_ = 26.6°, θ_min_ = 3.1°
CCD rotation images, thick slices scans	*h* = −13→13
Absorption correction: gaussian (Coppens *et al.*, 1965)	*k* = −18→20
*T*_min_ = 0.386, *T*_max_ = 0.613	*l* = −21→21
35034 measured reflections	

### Refinement

**Table:**

Refinement on *F*^2^	Primary atom site location: structure-invariant direct methods
Least-squares matrix: full	Secondary atom site location: difference Fourier map
*R*[*F*^2^ > 2σ(*F*^2^)] = 0.041	Hydrogen site location: inferred from neighbouring sites
*wR*(*F*^2^) = 0.109	H-atom parameters constrained
*S* = 1.09	*w* = 1/[σ^2^(*F*_o_^2^) + (0.0541*P*)^2^ + 8.7438*P*] where *P* = (*F*_o_^2^ + 2*F*_c_^2^)/3
6066 reflections	(Δ/σ)_max_ = 0.002
406 parameters	Δρ_max_ = 1.39 e Å^−^^3^
0 restraints	Δρ_min_ = −2.50 e Å^−^^3^

### Special details

**Table:**

Experimental. a grid of 8 x 8 x 8 = 512 sampling points was used in the absorption correction
Geometry. All e.s.d.'s (except the e.s.d. in the dihedral angle between two l.s. planes) are estimated using the full covariance matrix. The cell e.s.d.'s are taken into account individually in the estimation of e.s.d.'s in distances, angles and torsion angles; correlations between e.s.d.'s in cell parameters are only used when they are defined by crystal symmetry. An approximate (isotropic) treatment of cell e.s.d.'s is used for estimating e.s.d.'s involving l.s. planes.
Refinement. Refinement of *F*^2^ against ALL reflections. The weighted *R*-factor *wR* and goodness of fit *S* are based on *F*^2^, conventional *R*-factors *R* are based on *F*, with *F* set to zero for negative *F*^2^. The threshold expression of *F*^2^ > σ(*F*^2^) is used only for calculating *R*-factors(gt) *etc*. and is not relevant to the choice of reflections for refinement. *R*-factors based on *F*^2^ are statistically about twice as large as those based on *F*, and *R*- factors based on ALL data will be even larger.

### Fractional atomic coordinates and isotropic or equivalent isotropic displacement parameters (Å^2^)

**Table:**

	*x*	*y*	*z*	*U*_iso_*/*U*_eq_	
Re	0.804584 (18)	0.150099 (11)	0.635436 (10)	0.01716 (9)	
O1	0.5921 (4)	0.0300 (3)	0.5622 (2)	0.0328 (9)	
O2	0.9983 (4)	0.0058 (2)	0.6471 (2)	0.0302 (9)	
O3	0.8369 (4)	0.1791 (3)	0.46486 (19)	0.0268 (8)	
N1	0.7658 (4)	0.1315 (3)	0.7536 (2)	0.0199 (9)	
N2	0.6642 (4)	0.2458 (3)	0.6470 (2)	0.0193 (8)	
N3	0.9448 (4)	0.2463 (3)	0.6853 (2)	0.0196 (8)	
N4	1.3479 (5)	0.6998 (3)	0.9550 (3)	0.0292 (10)	
N5	0.7950 (6)	0.3895 (4)	0.8097 (4)	0.0460 (14)	
C1	0.6701 (5)	0.0737 (3)	0.5915 (3)	0.0225 (11)	
C2	0.9281 (5)	0.0614 (3)	0.6420 (3)	0.0234 (11)	
C3	0.8273 (5)	0.1710 (3)	0.5297 (3)	0.0244 (11)	
C4	0.8152 (5)	0.0726 (3)	0.8044 (3)	0.0237 (11)	
H4	0.8778	0.0375	0.7916	0.028*	
C5	0.7759 (6)	0.0619 (4)	0.8764 (3)	0.0304 (12)	
H5	0.8109	0.0193	0.9099	0.036*	
C6	0.6858 (6)	0.1141 (4)	0.8979 (3)	0.0278 (12)	
H6	0.6604	0.108	0.946	0.033*	
C7	0.6330 (5)	0.1772 (3)	0.8453 (3)	0.0221 (10)	
C8	0.5392 (5)	0.2344 (3)	0.8619 (3)	0.0228 (10)	
H8	0.5104	0.2306	0.9092	0.027*	
C9	0.4913 (5)	0.2947 (3)	0.8094 (3)	0.0228 (10)	
H9	0.4314	0.3322	0.8219	0.027*	
C10	0.5316 (5)	0.3013 (3)	0.7355 (3)	0.0206 (10)	
C11	0.4825 (6)	0.3620 (3)	0.6783 (3)	0.0255 (12)	
H11	0.4231	0.4012	0.6881	0.031*	
C12	0.5247 (6)	0.3614 (3)	0.6080 (3)	0.0260 (12)	
H12	0.4927	0.4001	0.5695	0.031*	
C13	0.6158 (5)	0.3028 (3)	0.5941 (3)	0.0215 (10)	
H13	0.6433	0.3039	0.5463	0.026*	
C14	0.6757 (5)	0.1834 (3)	0.7736 (3)	0.0182 (9)	
C15	0.6230 (5)	0.2453 (3)	0.7174 (3)	0.0189 (10)	
C16	0.9463 (5)	0.3204 (3)	0.6488 (3)	0.0225 (10)	
H16	0.8911	0.3283	0.6009	0.027*	
C17	1.0259 (5)	0.3845 (3)	0.6794 (3)	0.0241 (11)	
H17	1.0224	0.4346	0.6526	0.029*	
C18	1.1111 (5)	0.3747 (3)	0.7497 (3)	0.0222 (10)	
C19	1.1136 (5)	0.2970 (3)	0.7860 (3)	0.0229 (11)	
H19	1.1716	0.2868	0.8324	0.027*	
C20	1.0294 (5)	0.2353 (3)	0.7528 (3)	0.0212 (10)	
H20	1.0316	0.1844	0.7781	0.025*	
C21	1.1946 (5)	0.4445 (3)	0.7878 (3)	0.0237 (10)	
H21A	1.2319	0.4734	0.7484	0.028*	
H21B	1.2641	0.4223	0.8268	0.028*	
C22	1.1154 (6)	0.5050 (4)	0.8268 (4)	0.0398 (15)	
H22A	1.0503	0.5301	0.7868	0.048*	
H22B	1.072	0.4747	0.8624	0.048*	
C23	1.1966 (5)	0.5727 (4)	0.8718 (3)	0.0286 (13)	
C24	1.1984 (6)	0.6522 (3)	0.8408 (4)	0.0302 (13)	
H24	1.1498	0.6645	0.7917	0.036*	
C25	1.2736 (6)	0.7129 (3)	0.8839 (3)	0.0276 (12)	
H25	1.2727	0.7658	0.8624	0.033*	
C26	1.3481 (6)	0.6218 (4)	0.9832 (3)	0.0323 (13)	
H26	1.3998	0.6103	1.0315	0.039*	
C27	1.2757 (6)	0.5582 (4)	0.9441 (3)	0.0339 (13)	
H27	1.2798	0.5055	0.9662	0.041*	
C28	0.8459 (7)	0.3541 (4)	0.8642 (4)	0.0392 (16)	
C29	0.9137 (7)	0.3104 (5)	0.9339 (4)	0.0464 (16)	
H29A	0.9862	0.2814	0.9212	0.07*	
H29B	0.8565	0.2714	0.9513	0.07*	
H29C	0.9427	0.3498	0.975	0.07*	
P	0.72529 (14)	0.43615 (8)	0.41578 (7)	0.0227 (3)	
F1	0.6050 (3)	0.4560 (2)	0.45748 (19)	0.0332 (7)	
F2	0.7573 (3)	0.53361 (19)	0.41536 (18)	0.0299 (7)	
F3	0.8432 (4)	0.4168 (2)	0.3742 (2)	0.0422 (9)	
F4	0.6912 (4)	0.33928 (19)	0.4165 (2)	0.0399 (9)	
F5	0.6330 (3)	0.44566 (19)	0.33112 (17)	0.0294 (7)	
F6	0.8144 (4)	0.4265 (2)	0.50075 (19)	0.0373 (8)	

### Atomic displacement parameters (Å^2^)

**Table:**

	*U*^11^	*U*^22^	*U*^33^	*U*^12^	*U*^13^	*U*^23^
Re	0.01953 (14)	0.01844 (14)	0.01390 (12)	0.00022 (7)	0.00417 (8)	0.00007 (6)
O1	0.033 (2)	0.034 (2)	0.031 (2)	−0.0109 (18)	0.0033 (17)	−0.0095 (17)
O2	0.031 (2)	0.027 (2)	0.033 (2)	0.0107 (17)	0.0089 (17)	0.0046 (16)
O3	0.033 (2)	0.037 (2)	0.0114 (17)	−0.0021 (18)	0.0074 (14)	0.0034 (15)
N1	0.028 (2)	0.023 (2)	0.0108 (18)	0.0000 (18)	0.0081 (16)	0.0028 (16)
N2	0.024 (2)	0.017 (2)	0.0179 (19)	0.0026 (16)	0.0055 (16)	0.0011 (16)
N3	0.019 (2)	0.021 (2)	0.0177 (19)	−0.0012 (17)	0.0015 (16)	0.0001 (16)
N4	0.036 (3)	0.024 (2)	0.026 (2)	−0.003 (2)	0.0039 (19)	−0.0001 (19)
N5	0.043 (3)	0.043 (3)	0.049 (3)	−0.006 (3)	0.001 (3)	−0.008 (3)
C1	0.021 (3)	0.030 (3)	0.018 (2)	0.002 (2)	0.0066 (19)	−0.001 (2)
C2	0.027 (3)	0.027 (3)	0.017 (2)	−0.005 (2)	0.007 (2)	0.002 (2)
C3	0.018 (3)	0.019 (2)	0.034 (3)	−0.001 (2)	0.002 (2)	−0.001 (2)
C4	0.025 (3)	0.025 (3)	0.023 (2)	0.004 (2)	0.011 (2)	0.010 (2)
C5	0.046 (4)	0.027 (3)	0.020 (2)	0.009 (2)	0.009 (2)	0.007 (2)
C6	0.030 (3)	0.036 (3)	0.018 (2)	0.003 (2)	0.008 (2)	0.002 (2)
C7	0.021 (3)	0.028 (3)	0.017 (2)	−0.004 (2)	0.0031 (19)	−0.004 (2)
C8	0.026 (3)	0.026 (3)	0.018 (2)	−0.002 (2)	0.0067 (19)	−0.003 (2)
C9	0.022 (3)	0.026 (3)	0.022 (2)	0.000 (2)	0.008 (2)	−0.005 (2)
C10	0.022 (3)	0.016 (2)	0.024 (2)	−0.0013 (19)	0.0051 (19)	−0.0038 (19)
C11	0.028 (3)	0.027 (3)	0.021 (3)	0.007 (2)	0.002 (2)	−0.002 (2)
C12	0.024 (3)	0.025 (3)	0.028 (3)	0.002 (2)	0.003 (2)	0.007 (2)
C13	0.022 (3)	0.024 (3)	0.019 (2)	0.000 (2)	0.0039 (19)	0.005 (2)
C14	0.018 (2)	0.020 (2)	0.016 (2)	−0.0026 (19)	0.0016 (18)	−0.0006 (18)
C15	0.019 (2)	0.020 (2)	0.018 (2)	−0.0019 (19)	0.0054 (18)	−0.0004 (19)
C16	0.026 (3)	0.024 (3)	0.017 (2)	0.000 (2)	0.0020 (19)	0.003 (2)
C17	0.028 (3)	0.020 (3)	0.024 (3)	0.000 (2)	0.006 (2)	0.005 (2)
C18	0.021 (3)	0.021 (3)	0.026 (3)	0.001 (2)	0.009 (2)	−0.001 (2)
C19	0.023 (3)	0.027 (3)	0.017 (2)	−0.001 (2)	0.0006 (19)	0.002 (2)
C20	0.027 (3)	0.020 (2)	0.017 (2)	−0.001 (2)	0.0037 (19)	0.0004 (19)
C21	0.028 (3)	0.024 (3)	0.020 (2)	−0.003 (2)	0.006 (2)	−0.001 (2)
C22	0.025 (3)	0.034 (3)	0.061 (4)	−0.003 (3)	0.010 (3)	−0.019 (3)
C23	0.022 (3)	0.027 (3)	0.039 (3)	−0.004 (2)	0.012 (2)	−0.012 (2)
C24	0.031 (3)	0.032 (3)	0.026 (3)	0.007 (2)	0.000 (2)	−0.004 (2)
C25	0.031 (3)	0.023 (3)	0.029 (3)	0.000 (2)	0.005 (2)	0.000 (2)
C26	0.042 (4)	0.031 (3)	0.024 (3)	−0.001 (3)	0.007 (2)	0.002 (2)
C27	0.049 (4)	0.023 (3)	0.033 (3)	−0.002 (3)	0.018 (3)	0.006 (2)
C28	0.036 (4)	0.036 (4)	0.045 (4)	−0.009 (3)	0.007 (3)	−0.013 (3)
C29	0.045 (4)	0.048 (4)	0.045 (4)	−0.005 (3)	0.006 (3)	−0.005 (3)
P	0.0310 (8)	0.0219 (7)	0.0153 (6)	0.0034 (5)	0.0046 (5)	0.0005 (5)
F1	0.0371 (19)	0.0351 (18)	0.0312 (16)	0.0021 (15)	0.0162 (14)	−0.0018 (14)
F2	0.0366 (19)	0.0246 (17)	0.0267 (15)	−0.0045 (13)	0.0013 (13)	0.0017 (13)
F3	0.048 (2)	0.048 (2)	0.0354 (18)	0.0192 (18)	0.0200 (16)	0.0068 (16)
F4	0.075 (3)	0.0202 (17)	0.0249 (17)	0.0009 (16)	0.0113 (18)	−0.0003 (13)
F5	0.0396 (19)	0.0265 (16)	0.0184 (14)	−0.0059 (14)	−0.0035 (13)	0.0014 (12)
F6	0.045 (2)	0.040 (2)	0.0226 (16)	0.0098 (16)	−0.0042 (14)	0.0071 (14)

### Geometric parameters (Å, º)

**Table:**

Re—C1	1.929 (5)	C13—H13	0.93
Re—C2	1.927 (6)	C14—C15	1.432 (7)
Re—C3	1.923 (6)	C16—C17	1.375 (8)
Re—N1	2.186 (4)	C16—H16	0.93
Re—N2	2.179 (4)	C17—C18	1.384 (7)
Re—N3	2.208 (4)	C17—H17	0.93
O1—C1	1.131 (7)	C18—C19	1.399 (7)
O2—C2	1.158 (7)	C18—C21	1.504 (7)
O3—C3	1.156 (7)	C19—C20	1.387 (7)
N1—C4	1.334 (6)	C19—H19	0.93
N1—C14	1.363 (7)	C20—H20	0.93
N2—C13	1.330 (6)	C21—C22	1.526 (8)
N2—C15	1.372 (6)	C21—H21A	0.97
N3—C20	1.346 (6)	C21—H21B	0.97
N3—C16	1.353 (7)	C22—C23	1.513 (8)
N4—C25	1.348 (7)	C22—H22A	0.97
N4—C26	1.349 (8)	C22—H22B	0.97
N5—C28	1.148 (9)	C23—C27	1.390 (8)
C4—C5	1.400 (7)	C23—C24	1.391 (8)
C4—H4	0.93	C24—C25	1.388 (8)
C5—C6	1.376 (8)	C24—H24	0.93
C5—H5	0.93	C25—H25	0.93
C6—C7	1.410 (8)	C26—C27	1.379 (9)
C6—H6	0.93	C26—H26	0.93
C7—C14	1.405 (7)	C27—H27	0.93
C7—C8	1.425 (7)	C28—C29	1.466 (10)
C8—C9	1.363 (7)	C29—H29A	0.96
C8—H8	0.93	C29—H29B	0.96
C9—C10	1.431 (7)	C29—H29C	0.96
C9—H9	0.93	P—F3	1.587 (4)
C10—C15	1.403 (7)	P—F6	1.601 (3)
C10—C11	1.420 (7)	P—F4	1.603 (3)
C11—C12	1.375 (8)	P—F2	1.608 (3)
C11—H11	0.93	P—F5	1.611 (3)
C12—C13	1.404 (8)	P—F1	1.612 (3)
C12—H12	0.93		
			
C3—Re—C2	88.9 (2)	N3—C16—C17	122.9 (5)
C3—Re—C1	87.2 (2)	N3—C16—H16	118.6
C2—Re—C1	89.6 (2)	C17—C16—H16	118.6
C3—Re—N2	100.01 (19)	C16—C17—C18	120.4 (5)
C2—Re—N2	171.09 (18)	C16—C17—H17	119.8
C1—Re—N2	91.25 (19)	C18—C17—H17	119.8
C3—Re—N1	175.83 (19)	C17—C18—C19	116.9 (5)
C2—Re—N1	95.09 (19)	C17—C18—C21	122.2 (5)
C1—Re—N1	91.52 (19)	C19—C18—C21	120.9 (5)
N2—Re—N1	76.02 (16)	C20—C19—C18	119.9 (5)
C3—Re—N3	93.08 (19)	C20—C19—H19	120
C2—Re—N3	95.64 (19)	C18—C19—H19	120
C1—Re—N3	174.74 (19)	N3—C20—C19	122.6 (5)
N2—Re—N3	83.52 (16)	N3—C20—H20	118.7
N1—Re—N3	87.78 (16)	C19—C20—H20	118.7
C4—N1—C14	118.4 (4)	C18—C21—C22	110.2 (5)
C4—N1—Re	126.7 (4)	C18—C21—H21A	109.6
C14—N1—Re	114.8 (3)	C22—C21—H21A	109.6
C13—N2—C15	118.2 (4)	C18—C21—H21B	109.6
C13—N2—Re	127.4 (3)	C22—C21—H21B	109.6
C15—N2—Re	114.5 (3)	H21A—C21—H21B	108.1
C20—N3—C16	117.3 (4)	C23—C22—C21	112.6 (5)
C20—N3—Re	122.5 (3)	C23—C22—H22A	109.1
C16—N3—Re	120.2 (3)	C21—C22—H22A	109.1
C25—N4—C26	116.0 (5)	C23—C22—H22B	109.1
O1—C1—Re	176.6 (5)	C21—C22—H22B	109.1
O2—C2—Re	176.9 (5)	H22A—C22—H22B	107.8
O3—C3—Re	175.7 (5)	C27—C23—C24	117.0 (5)
N1—C4—C5	122.1 (5)	C27—C23—C22	122.1 (6)
N1—C4—H4	119	C24—C23—C22	120.8 (6)
C5—C4—H4	119	C25—C24—C23	119.3 (5)
C6—C5—C4	120.3 (5)	C25—C24—H24	120.4
C6—C5—H5	119.8	C23—C24—H24	120.4
C4—C5—H5	119.8	N4—C25—C24	124.0 (5)
C5—C6—C7	118.6 (5)	N4—C25—H25	118
C5—C6—H6	120.7	C24—C25—H25	118
C7—C6—H6	120.7	N4—C26—C27	123.5 (5)
C14—C7—C6	117.9 (5)	N4—C26—H26	118.3
C14—C7—C8	119.2 (5)	C27—C26—H26	118.3
C6—C7—C8	122.9 (5)	C26—C27—C23	120.2 (5)
C9—C8—C7	120.8 (5)	C26—C27—H27	119.9
C9—C8—H8	119.6	C23—C27—H27	119.9
C7—C8—H8	119.6	N5—C28—C29	178.5 (8)
C8—C9—C10	121.0 (5)	C28—C29—H29A	109.5
C8—C9—H9	119.5	C28—C29—H29B	109.5
C10—C9—H9	119.5	H29A—C29—H29B	109.5
C15—C10—C11	117.7 (5)	C28—C29—H29C	109.5
C15—C10—C9	119.2 (5)	H29A—C29—H29C	109.5
C11—C10—C9	123.0 (5)	H29B—C29—H29C	109.5
C12—C11—C10	118.5 (5)	F3—P—F6	91.2 (2)
C12—C11—H11	120.7	F3—P—F4	90.6 (2)
C10—C11—H11	120.7	F6—P—F4	89.6 (2)
C11—C12—C13	120.4 (5)	F3—P—F2	90.3 (2)
C11—C12—H12	119.8	F6—P—F2	90.64 (18)
C13—C12—H12	119.8	F4—P—F2	179.1 (2)
N2—C13—C12	122.3 (5)	F3—P—F5	89.95 (19)
N2—C13—H13	118.8	F6—P—F5	178.8 (2)
C12—C13—H13	118.8	F4—P—F5	90.05 (19)
N1—C14—C7	122.7 (5)	F2—P—F5	89.73 (17)
N1—C14—C15	117.1 (4)	F3—P—F1	179.6 (2)
C7—C14—C15	120.1 (5)	F6—P—F1	89.17 (19)
N2—C15—C10	122.9 (4)	F4—P—F1	89.5 (2)
N2—C15—C14	117.5 (4)	F2—P—F1	89.67 (18)
C10—C15—C14	119.5 (4)	F5—P—F1	89.68 (19)
			
C2—Re—N1—C4	−2.6 (5)	Re—N1—C14—C7	176.1 (4)
C1—Re—N1—C4	87.1 (5)	C4—N1—C14—C15	−179.1 (5)
N2—Re—N1—C4	178.0 (5)	Re—N1—C14—C15	−2.9 (6)
N3—Re—N1—C4	−98.1 (5)	C6—C7—C14—N1	−0.3 (8)
C2—Re—N1—C14	−178.5 (4)	C8—C7—C14—N1	179.5 (5)
C1—Re—N1—C14	−88.8 (4)	C6—C7—C14—C15	178.6 (5)
N2—Re—N1—C14	2.1 (3)	C8—C7—C14—C15	−1.6 (7)
N3—Re—N1—C14	86.0 (4)	C13—N2—C15—C10	0.7 (7)
C3—Re—N2—C13	−2.3 (5)	Re—N2—C15—C10	−179.1 (4)
C1—Re—N2—C13	−89.7 (5)	C13—N2—C15—C14	179.9 (4)
N1—Re—N2—C13	179.1 (5)	Re—N2—C15—C14	0.0 (6)
N3—Re—N2—C13	89.7 (4)	C11—C10—C15—N2	−1.3 (7)
C3—Re—N2—C15	177.6 (4)	C9—C10—C15—N2	178.2 (5)
C1—Re—N2—C15	90.1 (4)	C11—C10—C15—C14	179.6 (5)
N1—Re—N2—C15	−1.1 (3)	C9—C10—C15—C14	−1.0 (7)
N3—Re—N2—C15	−90.4 (3)	N1—C14—C15—N2	1.9 (7)
C3—Re—N3—C20	−139.5 (4)	C7—C14—C15—N2	−177.1 (5)
C2—Re—N3—C20	−50.3 (4)	N1—C14—C15—C10	−178.9 (5)
N2—Re—N3—C20	120.7 (4)	C7—C14—C15—C10	2.1 (7)
N1—Re—N3—C20	44.6 (4)	C20—N3—C16—C17	−2.7 (8)
C3—Re—N3—C16	41.9 (4)	Re—N3—C16—C17	176.0 (4)
C2—Re—N3—C16	131.1 (4)	N3—C16—C17—C18	1.2 (8)
N2—Re—N3—C16	−57.9 (4)	C16—C17—C18—C19	1.5 (8)
N1—Re—N3—C16	−134.0 (4)	C16—C17—C18—C21	−176.4 (5)
C14—N1—C4—C5	1.1 (8)	C17—C18—C19—C20	−2.5 (8)
Re—N1—C4—C5	−174.7 (4)	C21—C18—C19—C20	175.4 (5)
N1—C4—C5—C6	−1.6 (9)	C16—N3—C20—C19	1.6 (8)
C4—C5—C6—C7	1.1 (9)	Re—N3—C20—C19	−177.0 (4)
C5—C6—C7—C14	−0.2 (8)	C18—C19—C20—N3	1.0 (8)
C5—C6—C7—C8	−180.0 (5)	C17—C18—C21—C22	76.3 (7)
C14—C7—C8—C9	−0.1 (8)	C19—C18—C21—C22	−101.5 (6)
C6—C7—C8—C9	179.6 (5)	C18—C21—C22—C23	175.2 (5)
C7—C8—C9—C10	1.3 (8)	C21—C22—C23—C27	−75.2 (8)
C8—C9—C10—C15	−0.7 (8)	C21—C22—C23—C24	102.5 (7)
C8—C9—C10—C11	178.7 (5)	C27—C23—C24—C25	−2.5 (9)
C15—C10—C11—C12	1.4 (8)	C22—C23—C24—C25	179.7 (6)
C9—C10—C11—C12	−178.0 (5)	C26—N4—C25—C24	1.1 (9)
C10—C11—C12—C13	−1.0 (8)	C23—C24—C25—N4	0.8 (10)
C15—N2—C13—C12	−0.2 (8)	C25—N4—C26—C27	−1.4 (9)
Re—N2—C13—C12	179.6 (4)	N4—C26—C27—C23	−0.3 (10)
C11—C12—C13—N2	0.4 (8)	C24—C23—C27—C26	2.2 (9)
C4—N1—C14—C7	−0.1 (7)	C22—C23—C27—C26	−180.0 (6)

### Hydrogen-bond geometry (Å, º)

**Table:**

*D*—H···*A*	*D*—H	H···*A*	*D*···*A*	*D*—H···*A*
C13—H13···F4	0.93	2.47	3.377 (6)	166
C16—H16···F6	0.93	2.38	3.180 (6)	145
C11—H11···F5^i^	0.93	2.55	3.327 (6)	142
C12—H12···F1^i^	0.93	2.55	3.355 (6)	145
C5—H5···F1^ii^	0.93	2.52	3.382 (6)	154
C19—H19···F4^iii^	0.93	2.49	3.150 (6)	128
C20—H20···F5^iii^	0.93	2.45	3.317 (6)	154
C21—H21*A*···F5^iv^	0.97	2.53	3.486 (6)	168
C22—H22*B*···O1^v^	0.97	2.53	3.211 (8)	127

Symmetry codes: (i) −*x*+1, −*y*+1, −*z*+1; (ii) −*x*+3/2, *y*−1/2, −*z*+3/2; (iii) *x*+1/2, −*y*+1/2, *z*+1/2; (iv) −*x*+2, −*y*+1, −*z*+1; (v) −*x*+3/2, *y*+1/2, −*z*+3/2.
